# Thermopower scaling in conducting polymers

**DOI:** 10.1038/s41598-020-64951-z

**Published:** 2020-05-15

**Authors:** Morgan Lepinoy, Patrice Limelette, Bruno Schmaltz, François Tran Van

**Affiliations:** 1 GREMAN, UMR 7347 CNRS-INSA-Université de Tours, Parc de Grandmont, 37200 Tours France; 2 PCM2E, EA 6299, Université de Tours, Parc de Grandmont, 37200 Tours France

**Keywords:** Electronic properties and materials, Thermoelectrics, Polymers

## Abstract

By directly converting heat into electricity, thermoelectric effects provide a unique physical process from heat waste to energy harvesting. Requiring the highest possible power factor defined as *α*^2^*σ*, with the thermopower *α* and the electrical conductivity *σ*, such a technology necessitates the best knowledge of transport phenomena in order to be able to control and optimize both *α* and *σ*. While conducting polymers have already demonstrated their great potentiality with enhanced thermoelectric performance, the full understanding of the transport mechanisms in these compounds is still lacking. Here we show that the thermoelectric properties of one of the most promising conducting polymer, the poly(3,4-ethylenedioxythiophene) doped with tosylate ions (PEDOT-Tos), follows actually a very generic behavior with a scaling relation as *α* ∝ *σ*^−1/4^. Whereas conventional transport theories have failed to explain such an exponent, we demonstrate that it is in fact a characteristic of massless pseudo-relativistic quasiparticles, namely Dirac fermions, scattered by unscreened ionized impurities.

## Introduction

**Figure 1:**
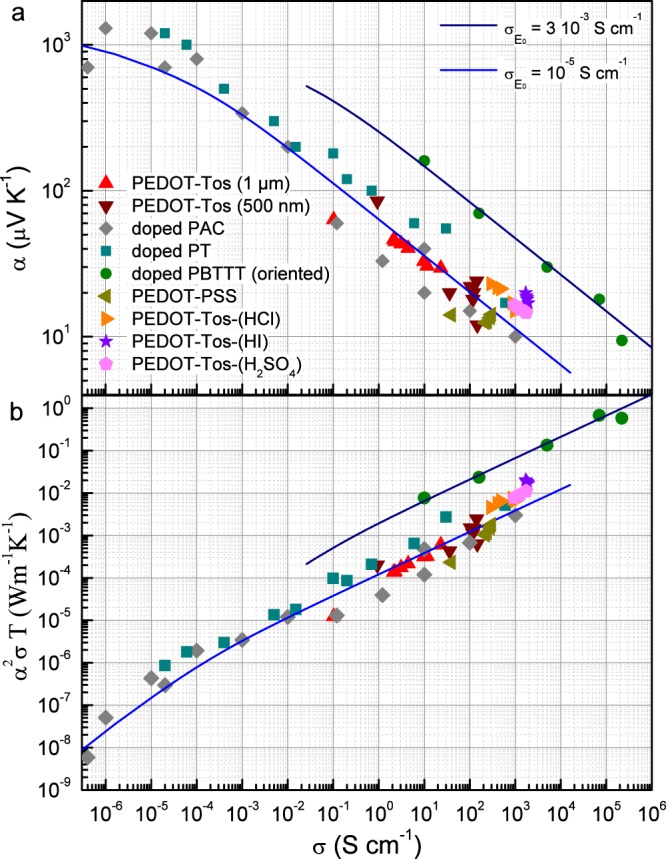
Scaling plots of the thermopower *α* (**a**) and the thermal power factor ${\alpha }^{2}\sigma T$ (**b**) as a function of the electrical conductivity *σ* at room temperature. The fitting lines have been calculated from the general expressions 4 leading to the power law scaling relation $\alpha \propto {\sigma }^{-1/4}$ (**a**) and accordingly ${\alpha }^{2}\sigma T\propto {\sigma }^{1/2}$ (**b**) in the degenerate limit, with ${\sigma }_{{E}_{0}}=3\,{10}^{-3}\,S\,c{m}^{-1}$ for the upper lines and ${\sigma }_{{E}_{0}}={10}^{-5}\,S\,c{m}^{-1}$ for the lower ones. Note that the departure from the power law dependence at low conductivity levels indicates a logarithmic scaling due to a non degenerate regime. While the PEDOT-Tos samples (500 nm and 1 *μ*m) refer to this work, the doped PAC are data from ref. ^8^, the doped PT (P3HT, PBTTT and P2TDC) are data from ref. ^10^, the oriented doped PBTTT are taken from ref. ^11^ and the PEDOT-PSS, PEDOT-Tos-(HCl), PEDOT-Tos-(HI) and PEDOT-Tos-(H_2_SO_4_) are data from refs. ^25–28^.

The understanding of both electrical and thermal transport phenomena in the conducting polymers is a key issue in order to be able to control and to optimize their properties^1–8^. Whereas the analysis of their electrical conductivity alone remains ambiguous likely due to their heterogeneous microstucture^8,9^, the growing interest for their thermoelectric properties^5–7^ allows now to compare the thermopower with the conductivity for a wide range of doping in several distinct compounds in order to find out some generic behaviors^2^. To our knowledge, Kaiser^8^ has first reported such an analysis in conducting polymers by collecting numerous data measured by several groups in polyacetylene samples (PAC) doped with different dopants and various levels of doping. By plotting the thermopower $\alpha$ as a function of the electrical conductivity $\sigma$ in a double log scale, he has shown a decrease of the former of two decades in amplitudes for an increase of the latter over eight decades. Despite some experimental dispersion in the data (some of them are displayed in Fig. 1a), these results already pointed out a power law behavior such as $\alpha \propto {\sigma }^{-1/4}$ even if this was not yet explicitly claimed. By investigating the impact of the doping method on conductivity and thermopower in polythiophenes (P3HT, PBTTT and P2TDC), Glaudell *et al*.^10^ uncovered the latter relationship and emphasized that it was not predicted by commonly used transport models. In an effort of clarifying the standard semi classical approach of transport phenomena including both electrical conductivity and thermopower, Kang and Snyder^2^ have demonstrated that a scaling law could relate in the degenerate limit the aforementioned transport coefficients such as $\alpha \propto {\sigma }^{-1/s}$. They claimed that the exponent s = 3 gave a superior fit to s = 4 and they highlighted that it was consistent with electrons scattered by unscreened ionized impurities in the frame of a three dimensional (3D) band model. Additionally, they noted a fundamentally different behavior in the previously reported data measured in PEDOT-Tos^7^ with an exponent s = 1. Very recently, new results have confirmed once more the unconventional scaling law with s = 4 between thermopower and conductivity in highly oriented polythiophene (P3HT, PBTTT) thin films^11^ as represented in Fig. 1a. Therefore, our reported measurements on thin films in Fig. 1a strongly suggest that the scaling law is indeed generic even in PEDOT-Tos samples for a wide range of doping.

## Results

Here two series of thin films have been investigated, each being characterized by samples with thickness of either 500 nm or 1 *μ*m (see the Methods section for the details). The former favors samples with higher electrical conductivity and lower thermopower accordingly while the latter ensures likely a better control of the doping as illustrated by the decrease of the fluctuations in Fig. 1a. Anyway, these two datasets agree with the overall reported scaling behavior and surprisingly even coincide quantitatively with the values measured in the other conducting polymers. The only sizable difference can be seen in the data of the highly oriented samples for which the electrical conductivity is typically enhanced from 2 or 3 orders of magnitude with respect to unoriented samples. Thus, the shift of the corresponding plot toward high conductivity values could result from their optimized microstruture and the overall data in Fig. 1a seem to reveal a highly generic behavior not only through the power law with a unique exponent s = 4 but also quantitatively.

On the other hand, the thermoelectric performance is usually quantified with the so called dimensionless figure of merit $ZT={\alpha }^{2}\sigma T/\kappa$, with the thermal conductivity $\kappa$. Another way to probe this efficiency is to consider the power factor, namely ${\alpha }^{2}\sigma$, or more usefully a thermal power factor ${\alpha }^{2}\sigma T$ which has the same unit than the thermal conductivity. Since $\kappa$ is of the order of $1\,W\,{m}^{-1}\,{K}^{-1}$ in most of the conducting polymers, and more frequently $\kappa \approx 0.5\,W\,{m}^{-1}\,{K}^{-1}$^7^, the thermal power factor is quantitatively representative of ZT as long as $\kappa$ remains of the order of $1\,W\,{m}^{-1}\,{K}^{-1}$. In agreement with the thermopower-conductivity relationship, the Fig. 1b demonstrates that the thermal power factor also follows a power law over several decades with conductivity with an exponent $\mathrm{1/2}$ due to the scaling exponent s = 4. In particular, it is sizably enhanced in oriented conducting polymers by approaching $1\,W\,{m}^{-1}\,{K}^{-1}$. Such a value highlights the potentiality of the conducting polymers in the context of thermoelectric applications even if thermal conductivity characterizations are required in order to precise ZT.

## Discussion

As previously emphasized, whereas semi classical transport model can account for the found general scaling form between thermopower and conductivity, the exponent s = 4 disagrees with conventional scattering mechanisms^10^. Therefore, we have re-investigated the latter in the frame of semi classical transport equations by considering either conventional electrons or Dirac fermions. This allows to recover the expressions of both the electrical conductivity and the thermopower in order to relate the so-called transport function^2^ introduced by Kang and Snyder to microscopic quantities. In addition, the origin of the exponent s can be discussed as well as the parameters influencing it. The general formulations of $\sigma$ and $\alpha$ can be deduced in terms of Fermi integrals and yield to scaling relations which hold in either non degenerate or degenerate regimes. Then, the relaxation time approximation allows to consider two scattering mechanisms for non relativistic and Dirac fermions. These calculations allow finally to discuss the different inferred exponents s consistent with the scaling behavior between $\alpha$ and $\sigma$ as observed experimentally in many conducting polymers.

The linearized semi-classical Boltzmann equation in presence of both thermal and chemical potential gradients in the frame of the relaxation time approximation accounts for the semi classical description of transport phenomena in solids. The x-component of the kinetic coefficients ${L}_{ij,x}$ and the transport coefficients, $\sigma$ and $\alpha$, are usually written according the following transport integrals^12,13^:

$${L}_{ij,x}=T\,\int \,{\tau }_{E}{v}_{x,E}^{2}{(E-\mu )}^{i+j-2}\left(\,,-,\frac{{\rm{\partial }}{f}_{E}}{{\rm{\partial }}E}\right){g}_{E}dE\,\sigma =\frac{{q}^{2}{L}_{11}}{T}\,\alpha =\frac{{L}_{12}}{qT{L}_{11}}$$

with the energy dependent relaxation time ${\tau }_{E}$, the velocity ${v}_{x,E}$, the density of states ${g}_{E}$ and the Fermi-Dirac statistic ${f}_{E}$, T being the temperature and $\mu$ the chemical potential. By noting $\theta$, $\nu$ and $\gamma$ the exponents of the relaxation time, the velocity and the density of states respectively, their energy dependence can be made explicit such as:

1 $${\tau }_{E}={\tau }_{0}{(E/{E}_{\tau })}^{\theta }\,{v}_{x,E}^{2}={v}_{0}^{2}{(E/{E}_{v})}^{\nu }\,{g}_{E}={g}_{0}{(E/{E}_{g})}^{\gamma }$$

Here, the characteristic energies ${E}_{\tau }$, ${E}_{v}$ and ${E}_{g}$, and the constants ${\tau }_{0}$, ${v}_{0}$ and ${g}_{0}$ have been introduced in order to focus on energy dependence. It follows that the kinetic coefficients can be written as:

$${L}_{ij,x}=T\frac{{\tau }_{0}{v}_{0}^{2}{g}_{0}}{{E}_{\tau }^{\theta }{E}_{v}^{\nu }{E}_{g}^{\gamma }}\,\int \,{E}^{\theta +\nu +\gamma }{(E-\mu )}^{i+j-2}\left(\,-\frac{\partial {f}_{E}}{\partial E}\right)dE$$

The electrical conductivity can then be expressed as an integral over quasiparticles reduced energy $\varepsilon =E/{k}_{B}T$.

2 $$\sigma ={q}^{2}\frac{{\tau }_{0}{v}_{0}^{2}{g}_{0}}{{E}_{\tau }^{\theta }{E}_{v}^{\nu }{E}_{g}^{\gamma }}\,\int \,{E}^{\theta +\nu +\gamma }\left(\,-\frac{\partial {f}_{E}}{\partial E}\right)dE=\int \,{\sigma }_{E}\left(\,-\frac{\partial {f}_{E}}{\partial E}\right)dE={\sigma }_{{E}_{0}}\,\int \,{\varepsilon }^{s}\left(\,-\frac{\partial {f}_{\varepsilon }}{\partial \varepsilon }\right)d\varepsilon $$

Thus, the relation used by Kang and Snyder^2^ is here recovered with the exponent $s=(\theta +\nu +\gamma )$ which combines the energy dependences exponents of the relaxation time, quasiparticles velocity and density of states. The Eq. 2 also relates explicitly the transport function introduced by these authors as ${\sigma }_{E}={\sigma }_{{E}_{0}}{\varepsilon }^{s}$ to microscopic quantities. Furthermore, the thermopower can be more conveniently rewritten below where the reduced chemical potential $\tilde{\mu }=\mu /{k}_{B}T$ has been introduced:

3 $$\alpha =\frac{q{L}_{12}}{{T}^{2}\sigma }=\frac{{k}_{B}}{q}\frac{{\sigma }_{{E}_{0}}}{\sigma }\,\int \,{\varepsilon }^{s}(\varepsilon -\tilde{\mu })\left(\,-\frac{\partial {f}_{\varepsilon }}{\partial \varepsilon }\right)d\varepsilon $$

As previously emphasized^2^, straightforward integrations by parts of Eqs. 2 and 3 allow to formulate the electrical conductivity and the thermopower as a function of Fermi integrals^14^ ${F}_{s}(\tilde{\mu })$.

4 $$\sigma ={\sigma }_{{E}_{0}}s{F}_{s-1}(\mathop{\mu }\limits^{ \sim })\,\alpha =\frac{{k}_{B}}{q}\left(\frac{(s+1){F}_{s}(\mathop{\mu }\limits^{ \sim })}{s{F}_{s-1}(\mathop{\mu }\limits^{ \sim })},-,\mathop{\mu }\limits^{ \sim }\right)\,with\,{F}_{s}(\mathop{\mu }\limits^{ \sim })={\int }_{0}^{{\rm{\infty }}}\,\frac{{x}^{s}}{{e}^{x-\mathop{\mu }\limits^{ \sim }}+1}dx$$

These formulations are general in the sense that they hold in both non-degenerate and degenerate cases. They allow in particular to relate the thermopower to the electrical conductivity through scaling relations in both regimes involving either logarithmic dependence in the non degenerate case^3,15^ or power law dependence in the degenerate one (see Supplementary Information for more details).

$$\alpha =\frac{{k}_{B}}{q}\left((,s,+,1),-,l,n,(\frac{\sigma }{{\sigma }_{{E}_{0}}\Gamma (s+1)})\right)\,or\,\alpha =\frac{{k}_{B}}{q}\frac{{\pi }^{2}}{3}s{\left(\frac{\sigma }{{\sigma }_{{E}_{0}}}\right)}^{-1/s}$$

While the logarithmic scaling form has been checked in less conducting polymers^4,11^ the observation of the power law scaling relation by several groups^8,10,11^ stresses that a degenerate regime is widely reached in the more conducting ones. This demonstrates that in these conducting polymers the Fermi energy lies above the transport edge at a microscopic level. We note that it has also recently been shown that strong disorder could imply departures from the latter power law^16^.

Since the exponent s results from the energy dependence of the relaxation time, quasiparticles velocity and density of states as $s=(\theta +\nu +\gamma )$, it has been argued that for conventional electrons the exponent of velocity is just $\nu =1$, due to the quadratic dependence of energy and the exponent of the density of state in 3 dimensions is $\gamma =1/2$ for free electrons. By considering the known scattering mechanisms, it has been noted^2^ that the exponent of the relaxation time due to unscreened ionized impurities scattering is $\theta =3/2$, which could successfully explain a scaling exponent s = 3 but disagrees with most of the experimental results^8,10,11^ as displayed in Fig. 1a. We also mention that the same exponent is expected for acoustic phonons scattering but in the low temperature regime, namely below the Debye temperature which is usually well below the room temperature in conducting polymers.

Furthermore, we have investigated the influence of the energy dispersion relation of quasiparticles on such a scaling, namely by considering that charge carriers are pseudo relativistic quasiparticles as Dirac Fermions. The most famous example of Dirac materials is very likely the graphene which consists in a single atomic layer of carbon with fascinating properties^17^. Its electronic structure is characterized by energy cones and if it is undoped, the Fermi energy is precisely located at the connection points of these cones which makes the graphene a 2D semi-metal^18^. Interestingly, the dispersion relation close to the Fermi level remains linear up to approximately 1 eV implying that the electronic excitations behave as Dirac Fermions, namely as massless pseudo relativistic quasiparticles^19^. As a consequence, the energy dispersion relation is ${E}_{k}={v}_{F}\hslash k$ and the Dirac fermions velocity is constant and equals the Fermi velocity ${v}_{F}$, thus the exponent $\nu =0$. In contrast, for D-dimensional non relativistic free fermions the energy is quadratic and ${v}_{x,E}^{2}={v}_{F}^{2}/D(E/{E}_{F})$, namely $\nu =1$. Therefore, in order to discuss the value of the scaling exponent s it is necessary to precise the density of states of Dirac fermions. By considering a spin degeneracy of 2 without valley degeneracy^18^, the density of states is declined below as a function of the D dimensionality of the quasiparticles.

$${g}_{1D,E}=\frac{2}{\pi \hslash {v}_{F}}\,{g}_{2D,E}=\frac{E}{\pi {(\hslash {v}_{F})}^{2}}\,{g}_{3D,E}=\frac{{E}^{2}}{{\pi }^{2}{(\hslash {v}_{F})}^{3}}$$

Note that the 2D density of states of graphene is here recovered with its linear energy dependence but without the usual factor 2 originating from its valley degeneracy^18^. Interestingly, the density of states can be reformulated by introducing the D-dimensional density of fermions ${n}_{D}$ and the Fermi energy ${E}_{F}$. This leads to a compact form of the Dirac (relativistic) fermions density of states ${g}_{D,E}^{r}$ which can be more easily compared to the non relativistic free fermions density of states ${g}_{D,E}^{nr}$.

$${g}_{D,E}^{r}=D\frac{{n}_{D}}{{E}_{F}}{\left(\frac{E}{{E}_{F}}\right)}^{D-1}\,{g}_{D,E}^{nr}=\frac{D}{2}\frac{{n}_{D}}{{E}_{F}}{\left(\frac{E}{{E}_{F}}\right)}^{\frac{D}{2}-1}$$

According to these relations, the density of states can be written with the exponent $\gamma$ equal to (D-1) for Dirac fermions and (D/2-1) for non relativistic fermions. It results that in the latter case the scaling exponent is $s=\theta +3/2$ whereas $s=\theta +2$ for Dirac fermions in 3D. So, semi classical calculations of relaxation time for Dirac fermions scattered by ionized impurities, unscreened and screened, or acoustic phonons, in the low and high temperatures regimes, lead to different $\theta$ exponents compared with those recovered for non relativistic fermions (see Supplementary Information for calculations details). Similar acoustic phonons scattering calculations have been carried out in 2D graphene^20^. Here, the inferred various scaling exponents summarized in the Table 1 demonstrate that the experimental scaling law between thermopower and electrical conductivity as $\alpha \propto {\sigma }^{-1/4}$ is accounted for by 3D Dirac fermions, namely Dirac fermions with parabolic density of states, scattered by unscreened ionized impurities. Quite recently, it has been shown that Mott-type Variable Range Hopping with a Gaussian density of states modified by Coulomb trapping of ionized dopants^21^ could reproduce approximately such a power law but only over a restricted range of conductivity, typically between 10^−3^ and ${10}^{-1}\,S\,c{m}^{-1}$. Whereas such a model may appear relevant in a low conductivity regime, it is inherently limited by its basic assumption of localized charge carriers. In contrast, our analysis successfully describes the thermopower-conductivity relationship over unrestricted conductivity regime, even reaching ${10}^{5}\,S\,c{m}^{-1}$ as displayed in Fig. 1a.

**Table 1:** Summary of the various parameters inferred from the calculated relaxation times including the deduced scaling exponents s for non relativistic and Dirac fermions.

*Quasiparticles*	*Scattering centers*	*τ*_0_	*E*_*τ*_	*θ*	*s* = *θ* + *γ* + *ν* (D = 3)
**Non relativistic fermions** ${E}_{k}={\hslash }^{2}{k}^{2}/2m$ $\nu =1$ $\gamma =D/2-1$	unscreened ionized impurities	$\frac{{a}_{i}/\,\sqrt{{E}_{i}\mathrm{/2}m}}{2\pi ln(si{n}^{-1}({\theta }_{min}/2))}$	${E}_{i}=\frac{Z{e}^{2}}{4\pi {\varepsilon }_{d}{a}_{i}}$	3/2	**3**
	screened ionized impurities	$\frac{{a}_{i}\,/\,(\hslash {k}_{0}\,/\,m)}{4\pi }{\left(\frac{{\hslash }^{2}{k}_{0}^{2}}{2m{E}_{i}}\right)}^{2}$	${\hslash }^{2}{k}_{0}^{2}\mathrm{/2}m$	−1/2	**1**
	Acoustic phonons $\frac{2k\hslash {v}_{s}}{{k}_{B}T}\gg 1$	$\frac{2\pi {(\hslash {v}_{s})}^{5}}{\zeta \mathrm{(5)}\Gamma \mathrm{(5)}}\frac{\sqrt{2m\,/\,{k}_{B}T}}{V{A}^{2}{({k}_{B}T)}^{3}}$	*k*_*B*_*T*	3/2	**3**
	Acoustic phonons $\frac{2k\hslash {v}_{s}}{{k}_{B}T}\ll 1$	$\frac{\pi {\hslash }^{4}{(\hslash {v}_{s}/{V}^{\mathrm{1/3}})}^{\mathrm{1/2}}}{2\sqrt{2}{A}^{2}{V}^{\mathrm{2/3}}{m}^{\mathrm{3/2}}{k}_{B}T}$	$\hslash {v}_{s}/{V}^{1/3}$	−1/2	**1**
**Dirac fermions** ${E}_{k}={v}_{F}\hslash k$ $\nu =0$ $\gamma =D-1$	unscreened ionized impurities	$\frac{{a}_{i}\,/\,{v}_{F}}{4\pi ln(si{n}^{-1}({\theta }_{min}/2))}$	${E}_{i}=\frac{Z{e}^{2}}{4\pi {\varepsilon }_{d}{a}_{i}}$	2	**4**
	screened ionized impurities	$\frac{{a}_{i}/{v}_{F}}{16\pi }{\left(\frac{{v}_{F}\hslash {k}_{0}}{{E}_{i}}\right)}^{2}$	${v}_{F}\hslash {k}_{0}$	−2	**0**
	Acoustic phonons $\frac{2k\hslash {v}_{s}}{{k}_{B}T}\gg 1$	$\frac{2\pi {(\hslash {v}_{s})}^{5}}{{v}_{F}{A}^{2}V{({k}_{B}T)}^{3}\zeta \mathrm{(5)}\Gamma \mathrm{(5)}}$	*k*_*B*_*T*	2	**4**
	Acoustic phonons $\frac{2k\hslash {v}_{s}}{{k}_{B}T}\ll 1$	$\frac{\pi {\hslash }^{3}{v}_{F}{v}_{s}}{2{A}^{2}{V}^{\mathrm{1/3}}{k}_{B}T}$	$\hslash {v}_{s}/{V}^{\mathrm{1/3}}$	−2	**0**

Note that *τ*_0_ and *E*_*τ*_ are not defined independently. The exponents *ν*, *γ* and *θ* refer to relations 1 while the definitions of the other parameters are provided in the Supplementary Information.

On the other hand, band structure calculations have been performed in PEDOT-Tos crystal by Kim and Brédas^22^ using GGA-DFT approximation for a crystal structure optimized leading to a space group $Pmn{2}_{1}$. The authors found a strong reduction of the gap due the tosylate doping and a metallic band structure which confirms degenerate fermionic states. This justifies the use of the thermopower scaling relation demonstrated in the degenerate regime in agreement with Fig. 1a. Furthermore, the calculated band structure displays dispersive branches crossing the Fermi level with pronounced linear behavior. Such a characteristic also appears in agreement at a qualitative level with the existence of Dirac fermions as the low energy quasiparticles. In addition, nearly crossing bands with extremely small gap have been predicted according these calculations along and perpendicular (*π* stacking) to the polymer chains in close analogy with Dirac cones. Nevertheless, due to the simulated position of the tosylate dopant in the structure, these authors have suggested an insulating behavior perpendicular to the PEDOT layers leading therefore to a two-dimensional-like metal. Since the latter prediction results mainly from the exact location and orientation of the tosylate ions which are not really supported by any experimental data^22,23^ an anisotropic 3D Dirac metal remains a plausible scenario consistent with the reported thermopower scaling analysis with the exponent s = 4. For instance, the observation of bulk Dirac fermions with a large out-of-plane anisotropy has already been reported in the topological Dirac semimetal Cd_3_As_2_^24^. Our both results and analysis make therefore an unexpected connection between Dirac materials and conducting polymers. Finally, due to the scaling relation the thermal power factor is expected to vary with the reduced chemical potential with the exponent s-2 as discussed in the Supplementary Information. Such a dependence demonstrates thus that the exponent s = 4 favors higher thermal power factor than s = 3, namely that Dirac fermions could favor better thermoelectric efficiency than conventional electrons.

## Methods

### Synthesis

PEDOT-Tos was synthesized by the chemical oxidative polymerization of 3,4-ethylenedioxythiophene following a previously described process^7^. The iron tosylate solution (40% in butanol) was purchased from Heraeus (Clevios-40), while EDOT and pyridine were purchased from Sigma-Aldrich. All materials were used as received.

Pyridine was added in the Fe(Tos)_3_ solution in order to reach a ratio of 0.5 mol of pyridine per 1 mol of iron tosylate. This oxidant solution was stirred for 24 h at 0 °C. The mixture was then cooling down to −30 °C. Afterwards the EDOT monomer was added to the oxidant solution with an oxidant-to-monomer ratio of 2.3:1. The mixture was stirred continuously at −30 °C for 3 h. The resulting solution was spin-coated on glass substrates previously washed with aqua regia then acetone in an ultrasonic bath. Once the mixture was spread on the glass, it was annealed on a hot plate at 70 °C during 20 mn to initiate the polymerization reaction by evaporating butanol and pyridine. In order to remove the remaining oxidant and the unreacted monomers, the films were washed several times with distilled water and removed from the substrates to be put back on substrates to avoid side effect and to improve homogeneity. The PEDOT-Tos films were further dried under primary vacuum at 50 °C during 24 h. Thereby we obtain PEDOT-Tos films with a thickness of 500 ± 10 nm with a spinning speed of 500 rpm and an acceleration of 100 rpm/s or 1 *μ*m ± 10 nm with a spinning speed of 100 rpm and an acceleration of 100 rpm/s. The reduction of PEDOT-Tos was done by immersion of the sample in ethanolamine alcoholic solutions of different concentrations (from pure ethanolamine to 10^5^ times diluted in ethanol) for 24 h then dried under primary vacuum at 50 °C for 24 h.

### Characterization

Polymers have been characterized with infrared spectroscopy, UV-Vis-NIR spectroscopy and X-ray diffraction.

Infrared analysis has been performed with a Perkin-Elmer Spectrum-One FTIR over the range of 1800–800 *cm*^−1^. All spectra show the typical bands for PEDOT-Tos with variations depending the concentration of the chemical treatment (Fig. S2 in the Supplementary Information). The persistence of the signal of tosylate chemical functions for all basic treatments shows that the tosylate is not removed from the films but only protonated and become p-toluenesulfonic acid. No remaining iron from Fe(Tos)_3_ was observed.

UV-Vis-NIR spectroscopy was performed with a Jasco V-670 spectrometer over the range of 300–1500 nm (Fig. S3 in the Supplementary Information). Upon reduction of ethanolamine, PEDOT-Tos looses positive charges and thus changes color from light blue to dark purple due to the gradual decrease of polaronic and bipolaronic optical transitions which appear around 880 nm and beyond 1500 nm. The UV-Vis-NIR spectroscopy allows to see the disappearance of the polaronic and bipolaronic peaks in favor of the appearance of a neutral chain peak at 585 nm. The appearance of the p-toluenesulfonic acid peak at 325 nm can be noticed for strongest reductions: counter-ions stay inside the material’s structure as their protonated form. The oxidation state can be related to the 585 nm peak intensity as previously described^7^. Hence, it is possible to control the oxidation level of PEDOT-Tos by controlling the concentration of the reducing agent in the treatment solution.

X-ray diffraction analysis were performed using a D8 Bruker diffractometer (XRD) with a Cu K*α* radiation source ($\lambda \approx 0.15409\,{\rm{nm}}$) over the 2*θ* range of 3–50°. The XRD patterns show crystalline peaks superimposed on a broad scattering background which is characteristic of crystalline regions within an amorphous medium (Fig. S4 in the Supplementary Information). A 2 peaks structure is found at 6.5° and 12.2° dependent on the reduction level. They appear better defined in the PEDOT-Tos as-synthesized rather than in those treated with high ethanolamine concentration. The decrease of the peaks seems to indicate besides the dedoping, an alteration of the nanostructural organization which leads to a film less crystallized. Note that a missing peak at 25° usually ascribed to the face to face interchain stacking distance between thiophene rings can be recovered in superimposed films from the same synthesis. This suggests a face-on organization of the chains towards the glass substrate.

In order to perform thermoelectric measurements on these films, 100 nm gold electrodes have been deposited through a mask to ensure low contact resistances by using silver paste. Both the electrical conductivity and the thermopower measurements have been performed under a maintained secondary vacuum (P < 10^−5^ mbar) using a four points configuration with a Physical Properties Measurements System from Quantum Design. Temperature rise of 1% has been applied in order to measure the thermopower and an electrical current of 10 *μA* has been used for electrical conductivity measurements. The current intensity has been chosen to both stay in ohmic conditions and minimize Joule effect in the material.

## Supplementary information

Supplementary Information.

## References

[1] Mahan GD, Sofo JO (1996). The best thermoelectric. Proceedings of the National Academy of Sciences.

[2] Kang SD, Snyder J (2017). Charge-transport model for conducting polymers. Nat. Mater..

[3] Fritzsche H (1971). A general expression for the thermoelectric power. Solid State Communications.

[4] Mateeva N, Niculescu H, Schlenoff J, Testardi LR (1998). Correlation of seebeck coefficient and electric conductivity in polyaniline and polypyrrole. J. Appl. Phys..

[5] Dubey N, Leclerc M (2011). Conducting polymers: Efficient thermoelectric materials. Polymer Phys..

[6] Leclerc M, Nadjari A (2011). Green energy from a blue polymer. Nat. Mater..

[7] Bubnova O (2011). Optimization of the thermoelectric figure of merit in the conducting polymer poly(3,4-ethylenedioxythiophene). Nat. Mater..

[8] Kaiser AB (2001). Electronic transport properties of conducting polymers and carbon nanotubes. Rep. Prog. Phys..

[9] Kaiser, A. B. Systematic conductivity behavior in conducting polymers: Effects of heterogeneous disorder. *Adv*. *Mater*. **13**, 927–941, 10.1002/1521-4095(200107)13:12/13<927::AID-ADMA927>3.0.CO;2-B (2001).

[10] Glaudell AM, Cochran JE, Patel SN, Chabinyc ML (2015). Impact of the doping method on conductivity and thermopower in semiconducting polythiophenes. Adv. Energy Mater..

[11] Vijayakumar V (2019). Bringing conducting polymers to high order: Toward conductivities beyond 10^5^ s cm^−1^ and thermoelectric power factors of 2 mw m^−1^ k^−2^. Adv. Energy Mater..

[12] Ziman, J. M. *Electrons and Phonons* (Oxford Univ. Press, 1960).

[13] Ashcroft, N. W. & Mermin, N. D. *Solid state physics* (Saunders College New York, 1976).

[14] Fistul, V. I. *Heavily doped semiconductors* (Plenum Press, 1969).

[15] Mott, N. F. & Davis, E. A. *Electronic processes in non-crystalline materials* (Oxford Univ. Press 2nd edn, 1979).

[16] Boyle CJ (2019). Tuning charge transport dynamics via clustering of doping in organic semiconductor thin films. Nature Commun..

[17] Geim AK, Novoselov KF (2007). The rise of graphene. Nat. Mater..

[18] Castro Neto AH, Guinea F, Peres NMR, Novoselov KS, Geim AK (2009). The electronic properties of graphene. Rev. Mod. Phys..

[19] Novoselov KS (2005). Two-dimensional gas of massless dirac fermions in graphene. Nature.

[20] Hwang EH, Das Sarma S (2008). Acoustic phonon scattering limited carrier mobility in two dimensional extrinsic graphene. Phys. Rev. B.

[21] Abdalla H, Zuo G, Kemerink M (2017). Range and energetics of charge hopping in organic semiconductors. Phys. Rev. B.

[22] Kim EG, Bredas J-L (2008). Electronic evolution of poly(3,4-ethylenedioxythiophene) (pedot): From the isolated chain to the pristine and heavily doped crystals. J. Am. Chem. Soc..

[23] Aasmundtveit KE (1999). Structure of thin films of poly(3,4-ethylenedioxythiophene). Synth. Met..

[24] Liu ZK (2014). A stable three-dimensional topological dirac semimetal Cd_3_As_2_. Nat. Mater..

[25] Liu C (2010). Highly conducting free-standing poly(3,4-ethylenedioxythiophene)/poly(styrenesulfonate) films with improved thermoelectric performances. Synth. Met..

[26] Ullah Z (2015). Acido-basic control of the thermoelectric properties of poly(3,4-ethylenedioxythiophene)tosylate (PEDOT-Tos). thin films. J. Mater. Chem. C.

[27] Wang J, Cai K, Song H, Shen S (2016). Simultaneously enhanced electrical conductivity and Seebeck coefficient in poly(3,4-ethylenedioxythiophene) films treated with hydroiodic acid. Synth. Met..

[28] Wang J, Cai K, Shen S (2014). Enhanced thermoelectric properties of poly(3,4-ethylenedioxythiophene) films treated with H_2_SO_4_. Org. Electron..

