# Supplementary material for: Thermopower scaling in conducting polymers
Source: Sci Rep. 2020 May 15;10:8086. doi: 10.1038/s41598-020-64951-z (PMC7229021; doi:10.1038/s41598-020-64951-z)
Supplement: Supplementary file 1 — Supplementary Information. [file 41598_2020_64951_MOESM1_ESM.pdf]

# Supplementary information

## Thermopower scaling in conducting polymers

M. Lepinoy[1,2], P. Limelette[1], B. Schmaltz[2] and F. Tran Van[2]
[1]GREMAN, UMR 7347 CNRS-INSA-Université de Tours, Parc de Grandmont, 37200 TOURS, FRANCE
[2]PCM2E, EA 6299, Université de Tours, Parc de Grandmont, 37200 TOURS, FRANCE

Several groups have pointed out a scaling relation between the thermopower α and the electrical conductivity σ in different conducting polymers[1-3] such as $\alpha \propto \sigma^{-1/4}$ in agreement with our presently reported results in PEDOT-Tos. While the detailed analysis previously performed by Kang and Snyder[4] accounts for a scaling relation as $\alpha \propto \sigma^{-1/s}$ with $s \leqslant 3$, we propose thereafter a microscopic semi classical approach which seems to explain the unconventional exponent s=4 observed in many conducting polymers. As previously emphasized, whereas semi classical transport model can account for the general found scaling form, the exponent s=4 disagrees with conventional scattering mechanisms[2,4]. Therefore, we present thereafter semi classical transport equations leading to the expressions of both the electrical conductivity and the thermopower in order to relate the so-called transport function introduced by Kang and Snyder[4] to microscopic quantities. This allows to precise the origin of the exponent s and the parameters influencing it. Thus, general formulations of σ and α can be deduced in terms of Fermi integrals and yield to scaling relations which hold in either non degenerate or degenerate regimes. The relaxation time approximation allows then to consider two scattering mechanisms for non relativistic fermions and massless pseudo-relativistic fermions, namely Dirac fermions. The ionized impurities scattering, either unscreened or screened, is first presented and then, the acoustic phonons scattering is considered in the low and high temperature regimes. These calculations allow to reveal the characteristic energy dependence of the related relaxation times required to infer the scaling exponents s between α and σ. The influence of the scaling exponent on the thermoelectric efficiency is then finally discussed. Samples characterizations are provided at the end.

## 1. Semi classical transport equations

As stressed in the main text, the linearized semi-classical Boltzmann equation in presence of both thermal and chemical potential gradients in the frame of the relaxation time approximation accounts for the semi classical description of transport phenomena in solids. The x-component of the kinetic coefficients $L_{ij,x}$ and the transport coefficients, σ and α, are usually written according the following transport integrals[5,6]:

$$L_{ij,x} = T \int \tau_E v_{x,E}^2 (E-\mu)^{i+j-2} \left( -\frac{\partial f_E}{\partial E} \right) g_E \, dE \qquad \sigma = \frac{q^2 L_{11}}{T} \qquad \alpha = \frac{L_{12}}{q T L_{11}}$$

with the energy dependent relaxation time $\tau_E$, the velocity $v_{x,E}$, the density of states $g_E$ and the Fermi-Dirac statistic $f_E$, T being the temperature and μ the chemical potential. By noting θ, ν and γ the exponents of the relaxation time, the velocity and the density of states respectively, their energy dependence can be made explicit such as :

$$\tau_E = \tau_0 \, (E/E_\tau)^\theta \qquad v_{x,E}^2 = v_0^2 \, (E/E_v)^\nu \qquad g_E = g_0 \, (E/E_g)^\gamma \tag{S1}$$

Here, the characteristic energies $E_\tau$, $E_v$ and $E_g$, and the constants $\tau_0$, $v_0$ and $g_0$ have been introduced in order to focus on energy dependence. It follows that the kinetic coefficients can be written as:

$$L_{ij,x}=T\frac{\tau_0 v_0^2 g_0}{E_\tau^\theta E_v^\nu E_g^\gamma}\int E^{\theta+\nu+\gamma}(E-\mu)^{i+j-2}\left(-\frac{\partial f_E}{\partial E}\right)dE$$

The electrical conductivity can then be expressed as an integral over quasiparticles reduced energy $\varepsilon=E/k_BT$.

$$\sigma=q^2\frac{\tau_0 v_0^2 g_0}{E_\tau^\theta E_v^\nu E_g^\gamma}\int E^{\theta+\nu+\gamma}\left(-\frac{\partial f_E}{\partial E}\right)dE=\int \sigma_E\left(-\frac{\partial f_E}{\partial E}\right)dE=\sigma_{E_0}\int \epsilon^s\left(-\frac{\partial f_\epsilon}{\partial \epsilon}\right)d\epsilon \qquad \text{(S2)}$$

Thus, the relation used by Kang and Snyder[4] is here recovered with the exponent $s=(\theta+\nu+\gamma)$ which combines the energy dependences exponents of relaxation time, quasiparticles velocity and density of states. The equation S2 also relates the transport function $\sigma_{E_0}$ introduced by these authors to microscopic quantities as:

$$\sigma_{E_0}=v_0^2 g_0 q^2 \tau_0\frac{(k_BT)^s}{E_\tau^\theta E_v^\nu E_g^\gamma}$$

Furthermore, the thermopower can be more conveniently rewritten below where the reduced chemical potential $\tilde{\mu}=\mu/k_BT$ has been introduced:

$$\alpha=\frac{qL_{12}}{T^2\sigma}=\frac{k_B}{q}\frac{\sigma_{E_0}}{\sigma}\int \epsilon^s(\epsilon-\tilde{\mu})\left(-\frac{\partial f_\epsilon}{\partial \epsilon}\right)d\epsilon \qquad \text{(S3)}$$

# 2. General formulations of electrical conductivity and thermopower

As already emphasized[4], straightforward integrations by parts of Eq.S2 and S3 allow to formulate the electrical conductivity and the thermopower as a function of Fermi integrals[7] $F_s(\tilde{\mu})$ .

$$\sigma=\sigma_{E_0} s F_{s-1}(\tilde{\mu}) \qquad \alpha=\frac{k_B}{q}\left(\frac{(s+1)F_s(\tilde{\mu})}{sF_{s-1}(\tilde{\mu})}-\tilde{\mu}\right) \qquad \textit{with} \qquad F_s(\tilde{\mu})=\int_0^\infty \frac{x^s}{e^{x-\tilde{\mu}}+1}dx \qquad \text{(S4)}$$

These formulations are general in the sense that they hold in both non-degenerate and degenerate cases as discussed thereafter.

## 2.1. Non degenerate regime

In materials in which the electronic excitations are characterized by an energy gap corresponding to the difference between the bottom of the conducting band and the top of the valence band $\Delta=E_C-E_V$, the electrons gas is non degenerate. Therefore, the reduced chemical potential can be considered as negative, with respect to $E_C$, and in particular proportional to half of the gap in a symmetric case for instance ( $\tilde{\mu}=-\Delta/2k_BT$ ). Assuming $|\tilde{\mu}|\gg 1$ , the denominator

of the Fermi integrals (Eq.S4) can be simplified by neglecting 1 which allows to write $F_s(\tilde{\mu})$ at leading order as a function of the well known Γ function:

$$F_s(\tilde{\mu}) \approx \Gamma(s+1) e^{\tilde{\mu}} \quad with \quad \Gamma(s+1) = \int_0^\infty x^s e^{-x} dx = s\Gamma(s)$$

As a result, a standard activated behavior is found for the electrical conductivity,

$$\sigma = \sigma_{E_0} \Gamma(s+1) e^{\tilde{\mu}} \;\rightarrow\; \sigma_{E_0} \Gamma(s+1) e^{-\Delta/2k_B T}$$

and the expression of the thermopower drastically simplifies by displaying the usual $1/k_B T$ temperature dependence as observed in insulators.

$$\alpha = \frac{k_B}{q}\left((s+1) - \tilde{\mu}\right) \;\rightarrow\; \frac{k_B}{q}\left((s+1) + \frac{\Delta}{2k_B T}\right)$$

If the reduced chemical potential is now related to the electrical conductivity, a logarithmic scaling relation between the thermopower and the conductivity can be found as theoretically early demonstrated by Mott[8].

$$\tilde{\mu} = \ln\left(\frac{\sigma}{\sigma_{E_0}\Gamma(s+1)}\right) \;\Rightarrow\; \alpha = \frac{k_B}{q}\left((s+1) - \ln\left(\frac{\sigma}{\sigma_{E_0}\Gamma(s+1)}\right)\right) \qquad \text{(S5)}$$

Note that such a scaling relation has already been checked experimentally[9] even quite recently in highly oriented conducting polymers in the direction perpendicular to the orientation process[3], namely in the less conducting direction.

## 2.2. Degenerate regime

In materials with no energy gap at the Fermi level, such as in metals, the electrons gas is degenerate if its density is high enough. This implies that the reduced chemical potential is now positive in contrast to the previous case. By performing a straightforward Sommerfeld expansion up to second order $(\tilde{\mu} \gg 1)$ , the Fermi integrals (Eq.S4) can now be approximated[7] as it follows:

$$F_s(\tilde{\mu}) \approx \frac{(\tilde{\mu})^{s+1}}{(s+1)} + \frac{\pi^2}{6} s (\tilde{\mu})^{s-1}$$

At leading order, $F_{s-1}(\tilde{\mu}) \approx (\tilde{\mu})^s / s$ and the electrical conductivity is simply related to the chemical potential with the exponent s:

$$\sigma = \sigma_{E_0} (\tilde{\mu})^s \;\Rightarrow\; \tilde{\mu} = \left(\frac{\sigma}{\sigma_{E_0}}\right)^{1/s} \qquad \text{(S6)}$$

Considering the first two terms of the previous expansion, the thermopower can also be deduced from Eq.S4 and recovers a usual form for a metal by writing $\tilde{\mu} = E_F / k_B T$ with the Fermi energy $E_F$.

$$\alpha = \frac{k_B}{q} \frac{\pi^2}{3} \frac{s}{\tilde{\mu}} \;\rightarrow\; \frac{k_B}{q} \frac{\pi^2}{3} s \frac{k_B T}{E_F} \qquad \text{(S7)}$$

By combining the expression of the reduced chemical potential in Eq.S6 with Eq.S7, it follows a power law relation between the thermopower and the electrical conductivity in agreement with the relation of Kang and Snyder[4]:

$$\alpha = \frac{k_B}{q}\frac{\pi^2}{3}s\left(\frac{\sigma}{\sigma_{E_0}}\right)^{-1/s} \tag{S8}$$

Contrasting with the first scaling relation in the non degenerate case (Eq.S5), the latter is a power law only involving the exponent s, namely depending on the sum of the energy dependences exponents of the relaxation time, the velocity and the density of states as s=(θ+ν+γ).

## 2.3. Thermopower scaling

According the general formulations (S4) of the thermopower and the electrical conductivity, two kinds of scaling behavior are expected depending on the either non degenerate or degenerate regimes. In the former case, a logarithmic scaling form (Eq.S5) relates α to σ, whereas in the latter α follows a power law with σ (Eq.S8). These two regimes are explicitly illustrated in Fig.S1. The double logarithmic scale in Fig.S1(a) highlights the power law scaling in the degenerate regime whereas the semi logarithmic scale in Fig.S1(b) reveals the logarithmic scaling form in the non degenerate one.

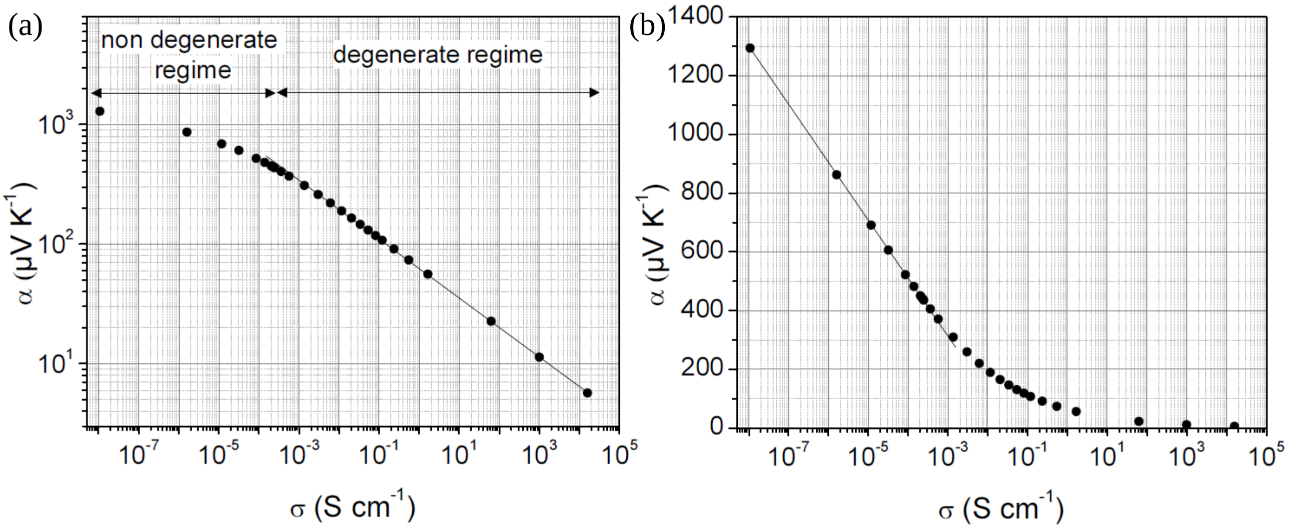


Figure S1: Scaling plot of the thermopower with the electrical conductivity as deduced from the general formulations in Eq.S4 where the Fermi integrals have been numerically calculated with s=4 and $\sigma_{E_0} = 10^{-5} S\ cm^{-1}$ . The two regimes are here clearly displayed with (a) the power law variation in agreement with Eq. S8 and (b) the logarithmic dependence at low conductivity levels according Eq.S5.

# 3. Relaxation time approximation

In order to determine the characteristic energy dependence in the transport integral, the relaxation time approximation is below briefly presented. The collision integral of the linearized Boltzmann equation is written as a function of the scattering rate $W_{k,k'}$ and the difference

between the first order correction $f^{(1)}_{k,k'}$ to the local equilibrium distribution $f^{(0)}_{k}$ considered as a Fermi-Dirac distribution[5].

$$\left(\frac{\partial f}{\partial t}\right)_{coll}=\frac{V}{(2\pi)^3}\int W_{kk'}(f^{(1)}_{k'}-f^{(1)}_{k})d^3k'\approx-\frac{f^{(1)}_{k}}{\tau(k)}$$

As far as we are concerned with a linear response regime, the out of equilibrium distribution $f^{(1)}_{k,k'}$ is proportional to the quasiparticles velocity and the applied perturbations formulated as gradients $\overrightarrow{\nabla X}$ of electrical potential or temperature for instance. Therefore, the relaxation time can be written as:

$$\frac{1}{\tau(k)}=\frac{V}{(2\pi)^3}\int W_{kk'}\left(1-\frac{f^{(1)}_{k'}}{f^{(1)}_{k}}\right)d^3k'=\frac{V}{(2\pi)^3}\int W_{kk'}\left(1-\frac{\vec{v}'.\overrightarrow{\nabla X}}{\vec{v}.\overrightarrow{\nabla X}}\right)d^3k'$$

Next, we consider that the scattering is here quasi elastic, namely there is no sizable change of the velocity modulus. By assuming $\vec{v}$ in the direction of a z axis, $\overrightarrow{\nabla X}$ in the xOz plane with an angle α with respect to the z axis, both velocity and gradient amplitudes cancel out. The expression of the relaxation time can be further simplified if the scattering rate does not depend on the basal angle ϕ, namely if it is only dependent on the scattering angle θ, as it will be case thereafter.

$$\frac{1}{\tau(k)}=\frac{V}{(2\pi)^3}\int W_{kk'}(1-(\cos\theta+\tan\alpha\sin\theta\cos\phi))d^3k'=\frac{V}{(2\pi)^3}\int W_{kk'}(1-\cos\theta)d^3k' \qquad \text{(S9)}$$

Finally, the scattering rate is given by the Fermi golden rule with the scattering potential $V_p$:

$$W_{kk'}=\frac{2\pi}{\hbar}|\langle k'|V_p|k\rangle|^2\delta(E_{k'}-E_k)$$

# 4. Ionized impurities scattering

Here we briefly review the main results leading to the conventional relaxation time for electrons scattered by ionized impurities[5,7] and then apply this formalism to Dirac fermions. In order to calculate the scattering rate, we need to precise the transition matrix elements between the two states k and k' due to the Coulomb interaction between quasiparticles and ionized impurities. From a general point of view, one can consider a screened Coulomb potential with the dielectric constant $\varepsilon_d$, the charge of the impurity Ze and the screening length $1/k_0$. The Fourier transform of the scattering potential leads then to the following expression.

$$V_p=\pm\frac{Ze^2}{4\pi\epsilon_d}\frac{e^{-k_0 r}}{r} \quad\Rightarrow\quad V_p(q)=\pm\frac{Ze^2}{\epsilon_d V}\frac{1}{(k_0^2+q^2)}$$

Here $q=|\vec{k}'-\vec{k}|\approx 2k'\sin(\theta/2)$ due to the quasi elastic interaction and the scattering rate can be finally written by using the fact that whatever the quasiparticles energy dispersion relation $\partial E_k/\partial k=\hbar v$ :

$$W_{kk'}=\frac{2\pi}{\hbar}\left(\frac{Ze^2}{\epsilon_d V}\frac{1}{(k_0^2+4k'^2\sin^2(\theta/2))}\right)^2\frac{\delta(k'-k)}{\hbar v'} \qquad \text{(S10)}$$

It follows that the relaxation time (Eq.S9) can be completely formulated as a function of the integration variables below.

$$\frac{1}{\tau(k)}=\frac{1}{\hbar^2 a_i^3(2\pi)^2}\left(\frac{Ze^2}{\epsilon_d}\right)^2\int\frac{(1-\cos\theta)\delta(k'-k)}{(k_0^2+4k'^2\sin^2(\theta/2))^2}\frac{k'^2}{v'}dk'\sin\theta d\theta d\varphi \quad \text{(S11)}$$

Note here that Eq.S9 has been multiplied by the number of ionized impurities $N_i$ and the mean distance between impurities $a_i$ has been introduced such as $N_i/V=1/a_i^3$.

## 4.1. Non relativistic fermions

The considered quasiparticles are here characterized by a quadratic dispersion relation such as $E_k=\hbar^2 k^2/2m$ with $\partial E_k/\partial k=\hbar^2 k/m=\hbar v$ as previously stated. Some basic algebra allows then to simplify the relaxation time in Eq.S11.

$$\frac{1}{\tau(k)}=\frac{m}{\hbar^3 a_i^3(2\pi)}\left(\frac{Ze^2}{\epsilon_d}\right)^2\int k\frac{4\cos(\theta/2)\sin^3(\theta/2)}{(k_0^2+4k^2\sin^2(\theta/2))^2}d\theta \quad \text{(S12)}$$

If the potential is very weakly screened, $k_0 << 1/a$ with the relevant atomic distance a, the previous integral is drastically simplified and reads:

$$\frac{1}{\tau(k)}=\frac{m}{\hbar^3 a_i^3(4\pi)k^3}\left(\frac{Ze^2}{\epsilon_d}\right)^2\int_{\theta_{min}}^{\pi}\frac{\cos(\theta/2)}{2\sin(\theta/2)}d\theta=\frac{m}{\hbar^3 a_i^3(4\pi)k^3}\left(\frac{Ze^2}{\epsilon_d}\right)^2\ln\left(1/\sin(\theta_{min}/2)\right)$$

One may emphasize that a minimum angle has been introduced above in order to take into account screening effect which removes the singularity at $\theta=0$ even if the screening is weak. So, the relaxation time can now be more conveniently written as a function of the energy:

$$\tau(E_k)=\frac{a_i^3(2mE_k)^{3/2}}{4\pi m\ln\left(1/\sin(\theta_{min}/2)\right)}\left(\frac{4\pi\epsilon_d}{Ze^2}\right)^2=\frac{a_i/\sqrt{E_i/m}}{\sqrt{2}\pi\ln\left(1/\sin(\theta_{min}/2)\right)}\left(\frac{E_k}{E_i}\right)^{3/2} \quad \text{(S13)}$$

where the potential-like energy $E_i=Ze^2/(4\pi\epsilon_d a_i)$ has been introduced for clarity.

If the potential is highly screened, $k_0 >> 1/a$, the k dependence in the denominator of Eq.S12 can be neglected:

$$\frac{1}{\tau(k)}=\frac{2\pi mk}{\hbar^3 k_0^4 a_i^3(2\pi)^2}\left(\frac{Ze^2}{\epsilon_d}\right)^2\int_0^{\pi}(1-\cos\theta)\sin\theta d\theta=\frac{16\pi m\sqrt{2mE_k}}{\hbar^4 k_0^4 a_i}\left(\frac{Ze^2}{4\pi\epsilon_d a_i}\right)^2$$

The relaxation time can now be written below as a function of the energy scale $\hbar^2 k_0^2/2m$ , the velocity $\hbar k_0/m$ and of course the quasiparticles energy $E_k$.

$$\tau(E_k)=\frac{a_i}{4\pi\hbar k_0/m}\left(\frac{\hbar^2 k_0^2/2m}{E_i}\right)^2\left(\frac{E_k}{\hbar^2 k_0^2/2m}\right)^{-1/2} \quad \text{(S14)}$$

Thus, depending on the screening strength the relaxation time is found to follow power law energy dependence with the conventional θ exponents 3/2 or -1/2.

## 4.2. Dirac fermions

Instead of conventional electrons, if the quasiparticles are Dirac fermions namely massless relativistic-like quasiparticles as observed in graphene for instance[10-12], the energy dispersion relation is now linear with a constant velocity $v_F$ such as $E_k = v_F \hbar k$ . This implies that the velocity in Eq.S11 is no more k-dependent and can be factorized. By using the potential-like energy $E_i$, the relaxation time can be simplified as:

$$\frac{1}{\tau(k)} = \frac{8\pi k^2}{\hbar^2 a_i v_F} E_i^2 \int \frac{4\cos(\theta/2)\sin^3(\theta/2)}{(k_0^2 + 4k^2 \sin^2(\theta/2))^2} d\theta \qquad \text{(S15)}$$

If the potential is very weakly screened, $k_0 << 1/a$, once again the angular integral is drastically simplified as:

$$\frac{1}{\tau(k)} = \frac{4\pi}{\hbar^2 a_i v_F k^2} E_i^2 \int_{\theta_{min}}^{\pi} \frac{\cos(\theta/2)}{2\sin(\theta/2)} d\theta = \frac{4\pi}{\hbar^2 a_i v_F k^2} E_i^2 \ln\left(1/\sin(\theta_{min}/2)\right)$$

In order to make the energy dependence more explicit, the relaxation time can be rewritten as it follows.

$$\tau(E_k) = \frac{a_i / v_F}{4\pi \ln\left(1/\sin(\theta_{min}/2)\right)} \left(\frac{E_k}{E_i}\right)^2 \qquad \text{(S16)}$$

If the potential is highly screened, $k_0 >> 1/a$, the k dependence in the denominator of Eq.S15 is neglected:

$$\frac{1}{\tau(k)} = \frac{16\pi k^2}{\hbar^2 k_0^4 a_i v_F} E_i^2 \int_0^{\pi} 2\cos(\theta/2)\sin^3(\theta/2) d\theta = \frac{16\pi k^2}{\hbar^2 k_0^4 a_i v_F} E_i^2$$

By introducing the quasiparticles energy $E_k$ and the energy scale $v_F \hbar k_0$ , the relaxation time becomes:

$$\tau(E_k) = \frac{a_i / v_F}{16\pi} \left(\frac{v_F \hbar k_0}{E_i}\right)^2 \left(\frac{E_k}{v_F \hbar k_0}\right)^{-2} \qquad \text{(S17)}$$

In contrast to non relativistic fermions, the energy dependence of the relaxation time is here characterized by the θ exponents 2 or -2 depending on screening.

# 5. Acoustic phonons scattering

The relaxation time of quasiparticles scattered by three dimensional acoustic phonons of wave vector q is still given by the integral in Eq.S9 with the scattering rate which now originates from the interaction Hamiltonian between quasiparticles and phonons. In contrast to the ionized impurities scattering, two process need here to be taken into account including the creation and the

annihilation of phonons[5]. By assuming a phonons occupation number $N_q$ high enough, the contributions of the aforementioned two process are nearly equal and the relaxation time can be written as:

$$\frac{1}{\tau(k)}=\frac{V}{(2\pi)^3}\int \frac{4\pi}{\hbar}|\gamma_q|^2 N_q \delta(E_{k+q}-E_k)(1-\cos\theta)d^3q \quad with \quad N_q=\frac{1}{e^{\hbar\omega_q/k_BT}-1}$$

In addition, considering the limit of long wave length acoustic phonons, the transition matrix element behaves as $|\gamma_q|^2=qA^2$ with the constant A which depends on atomic mass, sound velocity and interaction potential[5]. Finally, one must remember that the θ angle is the scattering one and not the angle between k and q, namely β. So, by noting that $(1-\cos\theta)=2\cos^2\beta$ , the relaxation time can be formulated as:

$$\frac{1}{\tau(k)}=\frac{A^2V}{\pi\hbar}\int N_q 2\cos^2(\beta)\delta(E_{k+q}-E_k)q^3\sin(\beta)dq\,d\beta \tag{S18}$$

## 5.1. Non relativistic fermions

If the quasiparticles energies have a quadratic dispersion relation, then the energy difference in Eq.S18 becomes $E_{k+q}-E_k=(\cos\beta+q/2k)\hbar^2kq/m$ and the relaxation time can be simplified below by taking into account the fact that $q/2k\leqslant 1$ .

$$\frac{1}{\tau(k)}=\frac{A^2Vm}{\pi\hbar^3k}\int N_q 2\cos^2(\beta)\delta\left(\left(\cos\beta+\frac{q}{2k}\right)\right)q^2\sin(\beta)dq\,d\beta=\frac{A^2Vm}{2\pi\hbar^3k^3}\int_0^{2k}q^4N_q dq$$

By considering the dimensionless variable $x=\hbar v_s q/k_BT$ the latter integral can be usefully reformulated such as:

$$\frac{1}{\tau(k)}=\frac{A^2Vm}{2\pi\hbar^3k^3}\left(\frac{k_BT}{\hbar v_s}\right)^5\int_0^{2k\hbar v_s/k_BT}x^4N_x dx \tag{S19}$$

Now, two regimes can be considered depending on the integral upper limit. If $2k\hbar v_s/k_BT\gg 1$ , the upper limit can be extended to infinite and the integral is approximated by a Bose one leading to the product of a Riemann ζ function and a gamma function:

$$\frac{1}{\tau(k)}=\frac{A^2Vm}{2\pi\hbar^3k^3}\left(\frac{k_BT}{\hbar v_s}\right)^5\zeta(5)\Gamma(5) \quad\Rightarrow\quad \tau(E_k)=\frac{2\pi}{\zeta(5)\Gamma(5)}\frac{(\hbar v_s)^5}{VA^2(k_BT)^3}\sqrt{\frac{2m}{k_BT}}\left(\frac{E_k}{k_BT}\right)^{3/2} \tag{S20}$$

If $2k\hbar v_s/k_BT\ll 1$ , the Bose factor can now be expanded as $N_x\approx 1/x$ and the integral in Eq.S19 leads to the following relaxation time.

$$\frac{1}{\tau(k)}=\frac{2A^2Vmk}{\pi\hbar^3}\left(\frac{k_BT}{\hbar v_s}\right)\Rightarrow\tau(E_k)=\frac{\pi\hbar^3}{2A^2Vmk}\left(\frac{\hbar v_s}{k_BT}\right)=\frac{\pi\hbar^4(\hbar v_s/V^{1/3})^{1/2}}{2\sqrt{2}A^2V^{2/3}m^{3/2}k_BT}\left(\frac{E_k}{\hbar v_s/V^{1/3}}\right)^{-1/2} \tag{S21}$$

Note here that the standard Bloch law is recovered in Eq.S20 with the $T^5$ power law dependence of $1/\tau$ at low temperatures followed by the T linear dependence at high temperatures in Eq.S21.

## 5.2. Dirac fermions

In the case of Dirac fermions, the energy difference in Eq.S18 is now $E_{k+q}-E_k=\hbar v_F k(\sqrt{1+(q/k)^2+(2q/k)\cos\beta}-1)$. By introducing the variable u=cos β and the function h(u) as the argument of the δ function, Eq.S18 becomes:

$$\frac{1}{\tau(k)}=\frac{2A^2V}{\pi\hbar^2 v_F k}\int N_q u^2\delta(h(u))q^3 dq\,du \quad with \quad u=(k/2q)\left((1+h(u))^2-(1+(q/k)^2)\right)$$

By taking into account the derivative $\partial u/\partial h(u)=(k/q)(1+h(u))$, the change of variable from u to h(u) can be achieved and the integral simplified by using the δ function and the dimensionless variable x as previously.

$$\frac{1}{\tau(k)}=\frac{A^2V}{2\pi\hbar^2 v_F k^2}\int_0^{2k} q^4 N_q dq=\frac{A^2V}{2\pi\hbar^2 v_F k^2}\left(\frac{k_B T}{\hbar v_s}\right)^5\int_0^{2k\hbar v_s/k_B T} x^4 N_x dx$$

If $2k\hbar v_s/k_B T\gg 1$, the integral can be extended and once more approximated by a Bose integral as the product of a Riemann ζ function and a gamma function:

$$\frac{1}{\tau(k)}=\frac{A^2V}{2\pi\hbar^2 v_F k^2}\left(\frac{k_B T}{\hbar v_s}\right)^5\zeta(5)\Gamma(5) \quad \Rightarrow \quad \tau(E_k)=\frac{2\pi(\hbar v_s)^5}{v_F A^2 V(k_B T)^3\zeta(5)\Gamma(5)}\left(\frac{E_k}{k_B T}\right)^2 \qquad \text{(S22)}$$

If $2k\hbar v_s/k_B T\ll 1$, the Bose factor can be expanded as $N_x\approx 1/x$ and the integral leads to the following relaxation time.

$$\frac{1}{\tau(k)}=\frac{2A^2Vk^2}{\pi\hbar^2 v_F}\left(\frac{k_B T}{\hbar v_s}\right) \quad \Rightarrow \quad \tau(E_k)=\frac{\pi\hbar^4 v_F^3}{2A^2V E_k^2}\left(\frac{\hbar v_s}{k_B T}\right)=\frac{\pi\hbar^3 v_F v_s}{2A^2V^{1/3}k_B T}\left(\frac{E_k}{\hbar v_F/V^{1/3}}\right)^{-2} \qquad \text{(S23)}$$

It is worth mentioning that due to the considered three dimensional acoustic phonons, the same temperature dependence as in the Bloch law is here found. In graphene for instance, it has been shown that similar calculations yield to a low temperature $T^4$ behavior due to the considered 2D phonons with the same energy dependence for the relaxation time[13].

# 6. Thermoelectric efficiency

The thermoelectric performance is usually quantified with the so called dimensionless figure of merit $ZT=\alpha^2\sigma T/\kappa$, with the thermal conductivity κ. Another way to probe this performance is to consider the power factor, namely $\alpha^2\sigma$, or more usefully a thermal power factor $\alpha^2\sigma T$ which has the same unit as the thermal conductivity. Since κ is of the order of 1 $Wm^{-1}K^{-1}$ in most of the conducting polymers, and more frequently $\kappa\approx 0.5\,W m^{-1}K^{-1}$ [14], the thermal power factor is quantitatively representative of ZT as long as κ remains of the order of unity.
By taking into account the scaling equation S8, the thermal power factor can be related to the electrical conductivity or equivalently to the reduced chemical potential.

$$\alpha^2 \sigma T = \left( \frac{k_B}{q} \frac{\pi^2}{3} s \right)^2 \left( \frac{\sigma}{\sigma_{E_0}} \right)^{1-2/s} \sigma_{E_0} T = \left( \frac{k_B}{q} \frac{\pi^2}{3} s \right)^2 (\tilde{\mu})^{s-2} \sigma_{E_0} T$$

Thus, the latter relation demonstrates that the thermal power factor is expected to scale with conductivity with an exponent ½ due to the scaling exponent s=4, as observed experimentally in Fig. 1b over several decades. It is worth mentioning that the thermal power factor is sizably enhanced in oriented conducting polymers by approaching 1 $Wm^{-1}K^{-1}$. This illustrates the potentiality of conducting polymers in the context of the thermoelectricity. The relation above between the thermal power factor and the reduced chemical potential with the exponent s-2 demonstrates also that the exponent s=4 favors higher thermal power factor than s=3, namely that Dirac fermions could favor better thermoelectric efficiency than conventional electrons.

# 7. Samples characterizations

Polymers have been characterized with infrared spectroscopy, UV-Vis-NIR spectroscopy and X-ray diffraction.

## 7.1. Infrared spectroscopy

Infrared analysis has been performed with a Perkin-Elmer Spectrum-One FTIR over the range of 1800-800 cm−1. All spectra in Fig.S2 show the typical bands for PEDOT-Tos with variations depending the concentration of the chemical treatment. The persistence of the signal of tosylate chemical functions for all basic treatments shows that the tosylate is not removed from the films but only protonated and become p-toluenesulfonic acid. No remaining iron from $Fe(Tos)_3$ was observed.

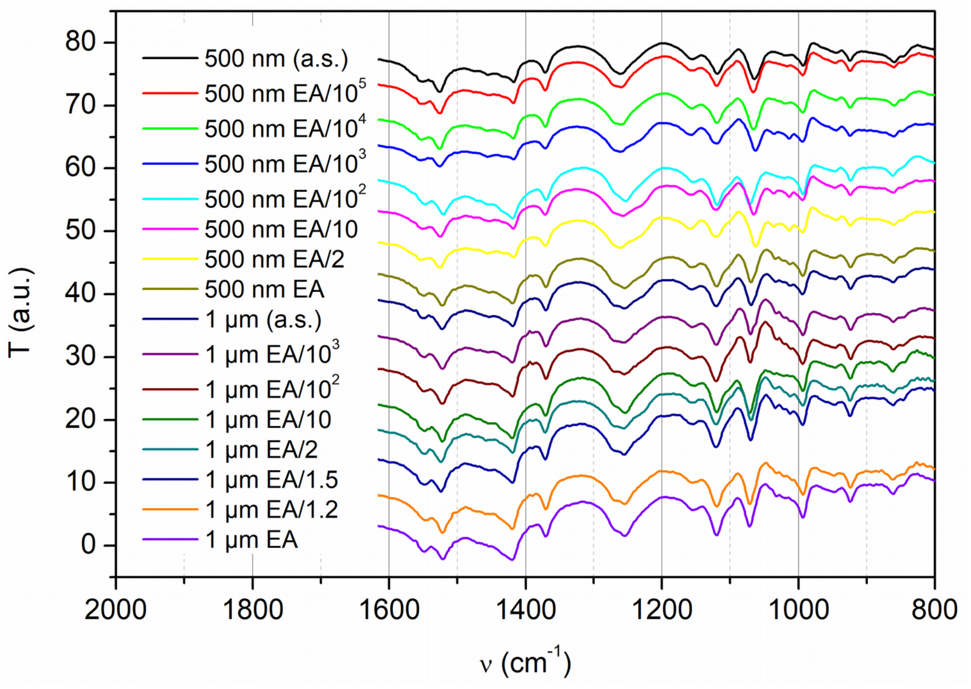


Figure S2: IR transmission spectra measured in the investigated PEDOT-Tos samples as synthesized (a.s.) and reduced with ethanolamine (EA).

## 7.2. UV-Vis-NIR spectroscopy

UV-Vis-NIR spectroscopy was performed with a Jasco V-670 spectrometer over the range of 300-1500 nm as displayed in Fig.S3. Upon reduction of ethanolamine, PEDOT-Tos looses positive charges and thus changes color from light blue to dark purple due to the gradual decrease of polaronic and bipolaronic optical transitions which appear around 880 nm and beyond 1500 nm. The UV-Vis-NIR spectroscopy allows to see the disappearance of the polaronic and bipolaronic peaks in favor of the appearance of a neutral chain peak at 585 nm. The appearance of the p-toluenesulfonic acid peak at 325 nm can be noticed for strongest reductions: counter-ions stay inside the material's structure as their protonated form. The oxidation state can be related to the 585 nm peak intensity as previously described[14]. Hence, it is possible to control the oxidation level of PEDOT-Tos by controlling the concentration of the reducing agent in the treatment solution.

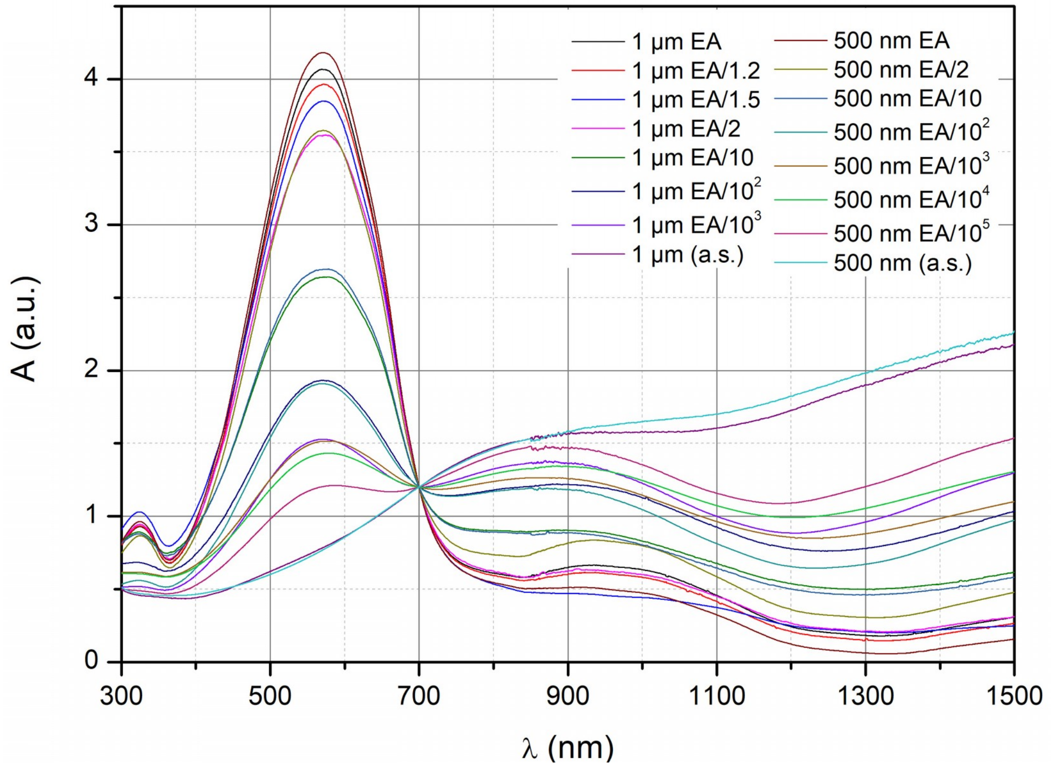


Figure S3: UV-Vis-NIR absorption spectra measured in the investigated PEDOT-Tos samples as synthesized (a.s.) and reduced with ethanolamine (EA).

## 7.3. X-ray diffraction

X-ray diffraction analysis were performed using a D8 Bruker diffractometer (XRD) with a Cu Kα radiation source (λ ≈ 0.15409 nm) over the 2θ range of 3-50°. The XRD patterns in Fig.S4 show crystalline peaks superimposed on a broad scattering background which is characteristic of crystalline regions within an amorphous medium. A 2 peaks structure is found at 6.5° and 12.2° dependent on the reduction level. They appear better defined in the PEDOT-Tos as-synthesized rather than in those treated with high ethanolamine concentration. The decrease of the peaks seems to indicate besides the dedoping, an alteration of the nanostructural organization which leads to a film less crystallized. Note that a missing peak at 25° usually ascribed to the face to face interchain

stacking distance between thiophene rings can be recovered in superimposed films from the same synthesis. This suggests a face-on organization of the chains towards the glass substrate.

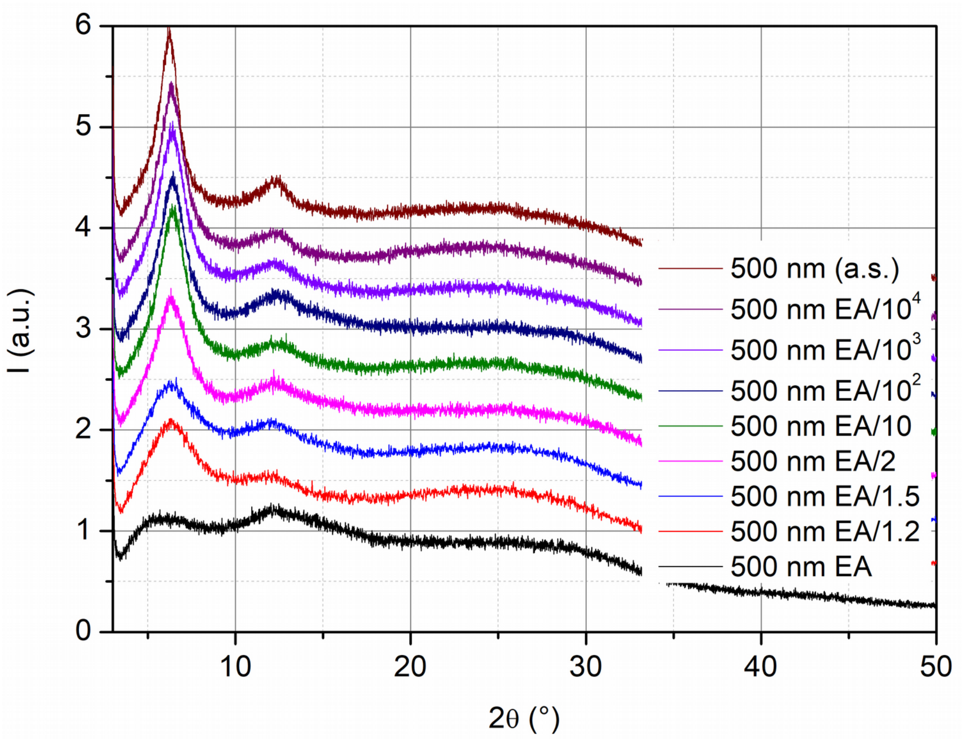


Figure S4: XRD patterns measured in the investigated PEDOT-Tos samples as synthesized (a.s.) and reduced with ethanolamine (EA).

## References


1. Kaiser, A. B. Electronic transport properties of conducting polymers and carbon nanotubes. *Rep. Prog. Phys.* **64**, 1-49 (2001).
2. Glaudell, A. M., Cochran, J. E., Patel, S. N. & Chabinyc, M. L. Impact of the Doping Method on Conductivity and Thermopower in Semiconducting Polythiophenes. *Adv. Energy Mater.* **5**, 1401072 (2015).
3. Vijayakumar, V. *et al.* Bringing Conducting Polymers to High Order: Toward Conductivities beyond $10^5$ S $cm^{-1}$ and Thermoelectric Power Factors of 2 mW $m^{-1}$ $K^{-2}$. *Adv. Energy Mater.* **9**, 1900266 (2019).
4. Kang, S. D. & Snyder, J. Charge-transport model for conducting polymers. *Nat. Mater.* **16**, 252-257 (2017).
5. Ziman, J. M. *Electrons and Phonons* (Oxford Univ. Press, 1960).
6. Ashcroft, N. W. & Mermin, N. D. *Solid State Physics* (Saunders College, New York, 1976).
7. Fistul, V. I. Heavily Doped Semiconductors Ch. 2 (Plenum Press, 1969).
8. Mott, N. F. & Davis, E. A. *Electronic Processes in Non-Crystalline Materials* 2nd edn (Oxford Univ. Press, 1979).
9. Mateeva, N., Niculescu, H., Schlenoff, J. & Testardi, L. R. Correlation of Seebeck coefficient and electric conductivity in polyaniline and polypyrrole. *J. Appl. Phys.* **83,** 3111–3117 (1998).
10. Geim, A. K. & Novoselov, K. F. The rise of graphene. *Nat. Mater.* ***6***, 183-191 (2007).
11. Castro Neto, A. H., Guinea, F., Peres, N. M. R., Novoselov, K. S. & Geim, A. K. The electronic properties of graphene. *Rev. Mod. Phys.* **81**, 109-162 (2009).

12. Novoselov, K. S. *et al.* Two-dimensional gas of massless Dirac fermions in graphene. *Nature* **438**, 197-200 (2005).
13. Hwang, E. H. & Das Sarma, S., Acoustic phonon scattering limited carrier mobility in two dimensional extrinsic graphene. *Phys. Rev. B* **77**, 115449-115454 (2008).
14. Bubnova, O. *et al.* Optimization of the thermoelectric figure of merit in the conducting polymer poly(3,4-ethylenedioxythiophene). *Nat. Mater.* **10**, 429-433 (2011).
